# A comparison between the effects of school‐based education programs provided by peer group versus health practitioners on premenstrual syndrome in adolescents: A protocol for a non‐masked clinical trial

**DOI:** 10.1002/nop2.858

**Published:** 2021-03-14

**Authors:** Farzaneh Babapour, Forouzan Elyasi, Jamshid Yazdani‐charati, Zohreh Shahhosseini

**Affiliations:** ^1^ Student Research Committee Mazandaran University of Medical Sciences Sari Iran; ^2^ Sexual and Reproductive Health Research Center, Psychiatry and Behavioral Sciences Research Center School of Medicine Addiction Institute Mazandaran University of Medical Sciences Sari Iran; ^3^ Health Sciences Research Center School of Health Mazandaran University of Medical Sciences Sari Iran; ^4^ Sexual and Reproductive Health Research Center Mazandaran University of Medical Sciences Sari Iran

**Keywords:** adolescent, health practitioner, peer educator, peer group, PMS, premenstrual syndrome, school‐based

## Abstract

**Aim:**

To compare the effect of school‐based education programs, by peer group versus health practitioner on Premenstrual Syndrome (PMS) in adolescent girls.

**Design:**

Non‐masked three‐armed clinical trial.

**Methods:**

Ninety 11th‐grade students with moderate to severe PMS will be allocated to Intervention Group 1 (IG1), Intervention Group 2 (IG2) and Comparison Group (CG). Three weeks (six online sessions) of parallel education will be implemented in IG1 by trained peer educators and in IG2 by a health practitioner. The primary outcome will be changes in PMS severity score between three groups over time (measured by Daily Record of Severity of Problems). Secondary outcomes include changes in Premenstrual Dysphoric Disorder and General Health. Data collection will be conducted in two‐time points, at baseline, and at the end of the intervention.

**Results:**

Our study will explore the effect of school‐based education programs, by peer group versus health practitioner on PMS. This will add to the evidence‐based interventions to PMS management and the effectiveness of peer education in health promotion of adolescents girls.

## 1 BACKGROUND

Premenstrual Syndrome (PMS) is an important health concern in adolescence that can significantly interfere with daily life activities and has a wide variety of physical and psychological signs and symptoms (Abeje & Berhanu, 2019; Mishra et al., 2015; Tadakawa et al., 2016). The American College of Obstetricians and Gynecologists (ACOG) has well‐established some criteria in describing PMS, including emotional symptoms (such as depression, angry outbursts, anxiety, irritability, crying spells, confusion, social withdrawal, poor concentration, insomnia, increased nap‐taking and changes in sexual desire) and physical symptoms (such as thirst and appetite changes, food cravings, breast tenderness, bloating and weight gain, headache, swelling of the hands or feet, aches and pains, fatigue, skin problems, abdominal pain and gastrointestinal symptoms). ACOG has also stated that the symptoms must be present in the 5 days before the menstrual cycle for at least 2–3 months, end within 4 days after the period starts, interfere with some of the females' normal activities, and not relapse until after day 12 of the menstrual cycle (ACOG Practice Bulletin, 2001). Although these symptoms may be not life‐threatening, they can seriously affect adolescents' learning activities as well as their mental health, interpersonal relationships and quality of life, that all of these are health‐related concerns worldwide (Buddhabunyakan et al., 2017; Delara et al., 2013; Kues et al., 2014). Existing literature has reported a 10% to 80% prevalence of PMS in adolescents worldwide and 40% to 80% in Iranian adolescents (Abeje & Berhanu, 2019; Delara et al., 2013; Ranjbaran et al., 2017; Tadakawa et al., 2016). Despite the high prevalence of PMS and the fact that adolescents with lower awareness about menstrual‐related problems showed a higher prevalence of menstrual disorders, their awareness about this issue is low (Seedhom et al., 2013).

Nowadays, for the management of PMS, various medical and non‐medical interventions are recommended (Appleton, 2018; Bahrami et al., 2018; Heydari et al., 2019; Maleki‐Saghooni et al., 2018; Tully et al., 2020; Yilmaz‐Akyuz & Aydin‐Kartal, 2019). Some studies have shown that education about changes in lifestyle; proper nutrition; and stress reduction techniques can be effective and even as the first step for PMS management (Aşcı et al., 2015; Steiner, 2000; Taghizadeh et al., 2010). One of the effective ways to educate adolescents is peer‐group education (Ghasemi et al., 2018). Peer education is a participatory teaching or sharing style in which people of similar social backgrounds, life experiences or group membership (peers) educate each other about specific topics (Southgate & Aggleton, 2016). One of the positive aspects of this method in adolescence is creating interaction between peers to exchange information since they spend most of their lives in schools and have a high impact on their classmates (Ghasemi et al., 2018; Lejone et al., 2020). Strengthening the critical thinking of adolescents, changing health attitudes, peers' good understanding from the surrounding social and cultural environment, and increasing self‐confidence are the other benefits of this approach (Seymour et al., 2013; Tolli, 2012). A well‐trained peer can communicate with others properly and effectively and in ways that the healthcare providers may be not able to do (Lotfi Mainbolagh et al., 2013). Therefore, replacing trained peers with healthcare providers in order to educate health‐related concepts for adolescents is a good opportunity (Campbell et al., 2014).

A review of the literature shows the effectiveness of peer education in HIV/AIDS prevention, sexual and reproductive health promotion, and drug and tobacco use cessation (Benton et al., 2020; Ghasemi et al., 2018; MacArthur et al., 2016; Sun et al., 2018). To the best of our knowledge, little is known about the effect of peer‐group education on PMS. If it is documented that educating adolescents by peers and health practitioner will have a similar effect on PMS, the available and cost‐effective peer educators can be used instead of the health practitioners. Therefore; this study aims to compare the effect of a school‐based education program by peer group versus health practitioner on PMS in adolescents.

### 1.1 Objectives and research hypothesis

The primary objective of this study is to compare the effectiveness of a school‐based education program by peer group versus health practitioner on PMS severity in adolescents that will be measured by Daily Records of Severity of Problems (DRSP). The secondary objectives are the effectiveness of the mentioned intervention on Premenstrual Dysphoric Disorder (PMDD) and General Health (GH) of the target group. The study has two main hypotheses: (1) We expect that education by peer group has a similar effect as education by health practitioner on PMS; (2) We expect the more reduction of PMS severity in intervention groups versus the comparison group.

## 2 METHODS/DESIGN

This protocol is based on Standard Protocol Items: Recommendations for Interventional Trials (SPIRIT) guidelines (Chan et al., 2013). (Additional file 1).

### 2.1 Study setting

This school‐based trial will be conducted in Sari city, the capital of Mazandaran province, northern Iran. This city consists of two urban districts and both of them have a separate Department of Education. In the Iranian educational system, schools divided into two main categories, Public schools (free) and Private schools (tuition). In this city, the majority of the high school students educated in public schools, and just about 19% of them study in private schools (Statistical Centre of Iran, 2016). This study will be conducted on students in public high schools from both districts in Sari city.

### 2.2 Study design

This study is a three‐armed parallel‐group non‐masked clinical trial, with two intervention groups named intervention group 1 (IG1), intervention group2 (IG2) and comparison group (CG). For prevention of contamination bias, school allocation to different arms will be random. Also, selection of students from the total eligible students in each school for participation in IGs and CG will be random. Outcomes assessment in IGs will occur at baseline (T0) and at the end of intervention as well as its equivalent in the comparison group (T1). After baseline assessment, the participants will be informed of which group they are assigned to.

### 2.3 Inclusion and exclusion criteria

The main target group is 11th‐grade public high school students who have completed a written informed consent form by themselves and their parents/guardians before the study begins. Inclusion criteria include (a) single; (b) moderate to severe PMS according to the Premenstrual Syndrome Screening Tool (PSST); (c) regular menstrual periods (21–35 days); and (d) not a professional athlete. Exclusion criteria include (a) use of therapies due to PMS, at baseline and during the study; (b) hormonal or psychiatric drug intake affecting PMS, at baseline and during the study; (c) diagnosis of a chronic disease; and (4) occurrence of unfortunate/stressful events for adolescents or their family in the last 6 months and during the study.

### 2.4 Intervention

**FIGURE 1:**
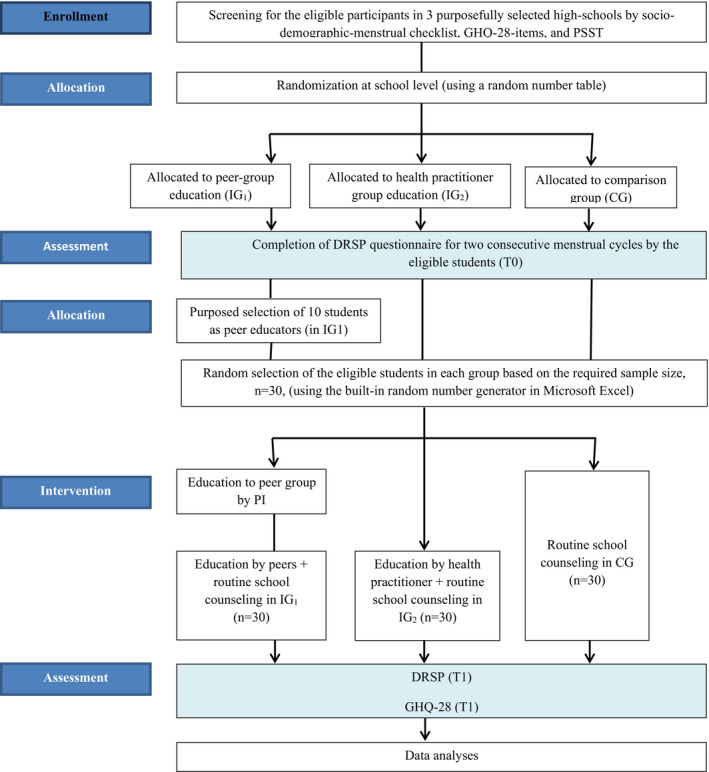
Study enrolment, allocation and intervention flow diagram

In this study, the following steps will be implemented (Figure 1):

- Schools will be purposefully selected from total schools in urban districts in Sari city;
- In each school, inclusion criteria will be assessed, including screening for moderate and severe PMS by PSST;
- After baseline assessment schools will be, randomly, allocated to one of three study arms: (a) Peer‐group education (IG1); (b) Health practitioner education (IG2); (c) comparison group (CG)
- All participants will be instructed to fill out the DRSP for two consecutive menstrual cycles;
- Re‐evaluation of exclusion criteria;
- Volunteer eligible students will be purposefully selected as peer educators and educated by the principal investigator (PI);
- The required sample for receiving interventions or participate as CG will be based on the random selection from the total eligible students in each school;
- Trained peer educators will implement the intervention in IG1;
- At the same time, the health practitioner will implement the intervention in IG2.

#### 2.4.1 Training of peer educators

At first, ten eligible volunteer students will be selected as potential peer educators to educate their peers. They will educate about life skills (include problem‐solving, critical thinking, effective communication skills, decision‐making, creative thinking, interpersonal relationship skills, self‐awareness building skills, empathy and coping with stress and emotions), puberty, menarche, physiology of menstruation, PMS, and its management (such as dietary changes and the use of aerobic exercise) by PI during six face‐to‐face 60‐min sessions, in 3 weeks (twice a week). In the following, six of them who are qualified from school staff as well as PI's point of view will be select as peer educators to implement the intervention. The PI is a Master of Science degree student in midwifery counselling.

##### Preference will be given to students who have demonstrated the following

- Assisting and contributing to a positive peer‐to‐peer environment;
- Have a history of student leadership/working with peers/groups in school;
- Enthusiasm and commitment for working on a team and interest in health topics;
- Ability to have effective communication skills, active listening, empathy, confidentiality, non‐judgmental listening and altruism;
- Availability to attend Peer Educator Training and meetings are mandatory.

#### 2.4.2 Peer‐group education

Participants in this group (IG1, *n* = 30, three subgroups with ten members) educated by two peer educators in each subgroup in six‐60‐min sessions for 3 weeks (twice a week) which will be supervised by the PI. The sessions will be in form of online in WhatsApp application and include voice records, audio PowerPoint, short video clips, educational pamphlets, and at the end of every session, sometime will be dedicated to question and answer. The content of sessions will include issues like puberty, menarche and menstrual cycles, PMS and its management.

#### 2.4.3 Health practitioner education

Simultaneously with the peer‐group education, participants in IG2 (*N* = 30) will be educated by a health practitioner in six‐60‐min sessions for 3 weeks (twice a week). The content and delivery route of each session will be similar in two intervention groups.

#### 2.4.4 Comparison group

The CG (*N* = 30), as well as IGs, will receive school routine education provided by school counsellors. In the Iranian educational system, in each school, a counsellor position has been defined that she usually answers students' questions in many different areas as well as menstrual concerns.

### 2.5 Sample size

Sample size will be determined at CI: 95% and Power: 80%, Large Cohen effect size = 0.8, in the intervention group, compared to the control group, and by the assistance of following formula:

$$n=\frac{2*{\left(z1‐\frac{\alpha }{2}+z1‐\beta \right)}^{2}}{E{\text{.S}}^{2}}=\frac{2*{\left(1.96+0.84\right)}^{2}}{{\left(0.8\right)}^{2}}=25.$$

z0.975=1.96z0.8=0.84.

By considering an attrition rate 25%, the sample size in each group will be calculated, 30 students.

### 2.6 Recruitment and randomization

Three high population public secondary high schools in both districts in Sari city will be selected purposefully. In each school, all 11th‐grade students will fill out the socio‐demographic menstrual characteristics checklist, GHQ‐28 Items, and PSST which is a retrospective questionnaire to screen eligible participants. After that, schools will be randomly allocated to one of three study arms: (a) Peer‐group education (IG1); (b) Health practitioner education (IG2); (c) Comparison Group (CG) by using a random number table. Following recruitment and verification of study eligibility, required sample size (30 students in each group) will be selected randomly by a random allocation sequence generating using the built‐in random number generator in Microsoft Excel (Cheusheva, 2020). They will complete the DRSP (during two consecutive menstrual cycles). Before random sampling, ten volunteer students will be selected as peer educators purposefully and will be trained by the PI. At the end of the final session, the PI will select six students as peer educators by assistance of school staff. Since the intervention is educational, it will not be possible to design a blind study. The students who meet the inclusion criteria will be informed completely about the protocol of the study.

### 2.7 Informed consent

School principals will provide informed consent on behalf of their school to participate in the study and will be introduced PI to school staff to collaborate with her. The PI will obtain written informed consent from each 11th‐grade student and their parents /guardians. Consent can be withdrawn at any time, without affects the student's relationship with the school staff and research team.

### 2.8 Outcomes

The primary outcome will be changes in PMS severity score between three groups over time that will be measured by DRSP. Secondary outcomes include changes in Premenstrual Dysphoric Disorder (by DRSP) and the General Health (by GHQ‐28 Items).

### 2.9 Data collection and outcome measurement

#### 2.9.1 Socio‐demographic‐menstrual characteristics checklist

The socio‐demographic‐menstrual characteristics checklist will fill out by the participants, which include following variables: Age, Marital status, Residential area (city/village), Parents’ education, Parents’ employment status, Height, Weight, Physical activity, Menarche age, Menstrual duration, Menstrual interval, Dysmenorrhea, Menstrual regularity and Familial history of PMS.

#### 2.9.2 Premenstrual symptoms screening tool (PSST)

The PSST was developed by Steiner et al. in 2003. It is a retrospective screening tool to identify women who suffer from severe PMS. This tool has 19 items in two parts, the first part measures 14 symptoms, including mood, physical and behavioural symptoms of PMS, and the second part (the last 5 items) measures the effect of these symptoms on a person's life. For each item, four possible responses (never, mild, moderate and severe) are considered from zero to three. The reliability of the PSST as measured by Cronbach's alpha coefficient is reported 0.93, and its content validity ratio and content validity index are reported 0.7 and 0.8, respectively (Hariri et al., 2013; Steiner et al., 2003, 2011).

#### 2.9.3 Daily record of severity of problems (DRSP)

Change in the PMS severity as a primary outcome is measured by the DRSP in three groups over time. The PMS severity will be prospectively evaluated in two‐time points, at baseline (T0) and after the intervention (T1) for two consecutive months (Mirghafourvand et al., 2015). The standard reference for PMS diagnosis based on the two cycles of DRSP charting is as follows: The luteal phase is defined as the 7 days before menses and the follicular phase as the 6 days after the onset of menses (Borenstein et al., 2007). The DRSP questionnaire developed by Endicott et al. and is a standard tool for determining the severity of PMS. It divided premenstrual symptoms according to DSM‐5 criteria into five areas: depression, anxiety, emotional, retention and physical symptoms (Epperson et al., 2012). This tool consists of 24 items (21 symptoms items and 3 dysfunctional items) and ranked from 0 (not at all) to 3 (extreme) (Endicott et al., 2006). The total score, from 7 days before until 4 days after period adds together and divided to the days' which incidence symptoms; in this way, we will calculate the mean severity of symptoms. This questionnaire's validity (by ten experts) and reliability (by test‐retest method) have been approved (Sharifi et al., 2014).

The PMDD will be assessed by the PSST which is the useful tool for DSM‐V PMDD. The PMDD causes severe irritability, depression or anxiety in a week before the period starts and usually goes away two to four days after the period starts (Biggs & Demuth, 2011). It will be assessed in two points, at baseline (T0) and at the end of two menstrual cycles after the intervention (T1).

#### 2.9.4 General health questionnaire 28 items (GHQ‐28)

Change in GH score is assessed by GHQ‐28 in two points, at baseline and at the end of two menstrual cycles after the intervention. The GHQ‐28 was developed by Goldberg and Hillier in 1979 and is based on Exploratory Factor Analysis of the original version of GHQ‐60 (Goldberg & Hillier, 1979). In the form of the GHQ‐28, items were selected to cover four main domains: somatic symptoms, anxiety and insomnia, social dysfunction and severe depression. Each item is accompanied by four possible responses and Likert scale scoring of four values (zero to three). The total score ranging from 0 to 84 and lesser score indicates better GH. It has been used in several studies (Abootalebi et al., 2020; Abuzied & Ali, 2020; Ebrahimi et al., 2007).

### 2.10 Statistical analysis

All analyses of the outcomes will performed as intention‐to‐treat analyses and following the TREND checklist. The normality of distribution will be examined by the Kolmogorov–Smirnov test. All quantitative variables will be expressed as mean ± *SD*. Categorical variables will be presented as frequencies and percentages. The chi‐square test will be used to test for differences between categorical variables. Baseline means differences will be tested using a one‐way analysis of variance (ANOVA or the non‐parametric equivalent of that Kruskal–Wallis). Analysis of covariance (ANCOVA) will be used to identify differences between the three groups at the end of the study, adjusting for baseline values and covariates. The comparison of mean values will be conducted within groups after the intervention period using paired sample *t* tests. *p* < .05 will be considered statistically significant. Statistical tests of the outcome measures will be performed using Statistical Package for the Social Sciences (SPSS version 18.0).

### 2.11 Ethics approval

This protocol approved by the Medical Ethics Committee of the Mazandaran University of Medical Sciences (MAZUMS) (approval number: IR.MAZUMS.REC.1396.9188) and in Iranian Registry of Clinical Trials (Code: IRCT20150608022609N5).

## 3 DISCUSSION

To the best of our knowledge, this is the first trial that will investigate the effectiveness of a school‐based peer‐group education versus health practitioner on PMS in adolescents. This study aims to address several gaps in the current scientific evidence for educational intervention and optimal use of adolescent's capabilities. Firstly, until now there have been a limited number of studies that investigate the effectiveness of peer‐group education on PMS, particularly in adolescents. Secondly, this comparison study between peer educators and health practitioner aims to use peer educators as an alternative to a health practitioner in areas with less access to healthcare providers in schools. This study evaluates the response of education by two different trainers, peer group and health practitioner, aims to use peer group instead of health practitioner to improve peers' ability on PMS management in the adolescents. To conclude, if the peer education intervention improves PMS, there is potential for the program to be widely implemented by schools resulting in a saves time and cost which is needed for healthcare providers training. Finally, with the utilization of peer‐group education, we will have a positive impact on adolescent population health.

### 3.1 Trial status

The trial was launched on 4 April 2020 and participant recruitment is still ongoing (Table 1).

**TABLE 1:** Timeline of the study; we expect 12 months for this trial

Explanation of research activities	Duration (month)											
	1	2	3	4	5	6	7	8	9	10	11	12
Enrolment and baseline assessment (T0)	*	*	*	*								
Recruitment and intervention					*	*						
Postintervention assessment (T1)							*	*				
Statistical analysis									*	*		
Writing the final report of the intervention											*	*
The expected time	*	*	*	*	*	*	*	*	*	*	*	*

## CONFLICT OF INTEREST

The authors declare that they have no competing interests.

## AUTHORS' CONTRIBUTION

ZS and FE: Conceptualization, design study and draft manuscript. FB: Project Coordinator for the study, sole PI of the study and assist in draft manuscript. JYC: Participation in the design of the study. All authors read and approved the final manuscript.

## Data Availability

The data of this study are available upon the journal request.
